# Pharmacodynamic and Pharmacokinetic Properties of Full Phosphorothioate Small Interfering RNAs for Gene Silencing *In Vivo*

**DOI:** 10.1089/nat.2020.0852

**Published:** 2021-06-04

**Authors:** Christian Berk, Gianluca Civenni, Yuluan Wang, Christian Steuer, Carlo V. Catapano, Jonathan Hall

**Affiliations:** ^1^ Department of Chemistry and Applied Biosciences, Institute of Pharmaceutical Sciences, ETH Zurich, Zurich, Switzerland.; ^2^ Institute of Oncology Research (IOR), Università della Svizzera Italiana (USI), Bellinzona, Switzerland.

**Keywords:** phosphorothioate, siRNA, backbone, delivery, stability

## Abstract

State-of-the-art small interfering RNA (siRNA) therapeutics such as givosiran and fitusiran are constructed from three variable components: a fully-modified RNA core that conveys metabolic stability, a targeting moiety that mediates target-cell uptake, and a linker. This structural complexity poses challenges for metabolite characterization and risk assessment after long-term patient exposure. In this study, we show that basic phosphorothioate modification of a siRNA targeting the oncoprotein Lin28B provides a useful increase in metabolic stability, without greatly compromising potency. We found that its stability *in vitro* matched that of nanoparticle-free patisiran in serum and surpassed it in liver tritosome extracts, although it did not reach the stability of the fitusiran siRNA core structure. Liver and kidney were the main sites of accumulation after its subcutaneous administration in mice. Despite the lack of a delivery agent-free antitumor effect, we anticipate our study to be a starting point to develop alternative siRNA scaffolds that can be degraded into naturally-occurring metabolites and help alleviate the aforementioned challenges. Furthermore, Lin28B is a promising target for cancers, and the development of such simplified siRNA analogs, possibly together with novel targeting units, holds potential.

## Introduction

Therapeutic oligonucleotides have emerged as a third pillar of modern pharmacotherapy, expanding the portfolio of traditional small molecule drugs and biologics [1,2]. A key advantage of this new drug class is its inherent potential to regulate the expression of any gene of interest through the rational design of a complementary oligonucleotide, including disease-causing genes that have been previously considered undruggable. The relatively straightforward design and predictable pharmacokinetic properties of oligonucleotides have enabled the development of a customized treatment for a single patient in <1 year [3], delivering on the promise of personalized medicine.

Small interfering RNAs (siRNAs) are one of the primary modalities in the field of RNA therapeutics, harnessing the endogenous RNA interference machinery for pharmacological interventions. In its classical format, siRNAs are duplexes of 21 nucleotide (nt) RNA strands with a 19 nt complementary stem and a 2 nt overhang on the 3′-end of each strand [4]. Several groups have reported the use of a diverse set of chemically modified siRNAs for *in vivo* silencing [5–11]. The clinical potential of this approach has been demonstrated through the recent approval of two siRNA therapeutics, patisiran [12,13] and givosiran [14]. Patisiran is only modestly chemically modified and therefore depends on its lipid nanoparticle (LNP) encapsulation for nuclease protection and efficient tissue delivery. However, givosiran is administered as a nanoparticle-free solution. For this purpose, this siRNA relies on complex chemical modification of the natural oligoribonucleotide in three main structural ways: (1) modification of every nucleoside with 2′-OMe or 2′-F moieties and terminal phosphorothioate (PS) linkages, (2) conjugation through a binary linker to (3), a trivalent *N*-acetylgalactosamine ligand for targeted delivery to hepatocytes [15]. The structural complexity of such molecules poses an additional challenge in terms of metabolite characterization and risk assessment with respect to long-term exposure.

As a minimalistic alternative to this complexity, we are exploring full PS substitution of otherwise unmodified siRNAs to increase their stability against nucleases, to extend their lifetime *in vivo*, and their delivery into target cells. While unconjugated phosphodiester (PO) siRNAs are known to be rapidly degraded and/or cleared from circulation by renal filtration [16–20], single stranded PS oligonucleotides are protected and shielded against urinary excretion due to their binding to serum proteins [21]. In fact, PS substitution was considered for the use in siRNA therapeutics early on, but was largely dismissed due to reports on reduced potency [22,23] and in some [24,25] but not all cases toxicity [26]. However, the behavior of cultured cells often does not reflect the *in vivo* situation, particularly in light of several reports highlighting the contribution of delivery agents to cellular toxicity [27,28], indicating that naked injection of synthetic siRNAs is less likely to trigger an immune response than LNP encapsulated siRNAs [29]. Moreover, full PS substitution of the passenger strand or the inclusion of single stranded PS tails in combination with full 2′-modification and an asymmetric siRNA design have been recently reported to favor a broad biodistribution without causing significant toxicity *in vivo* [19,30,31].

We have previously shown that the silencing activity of PS siRNAs is almost equal to that of their PO counterparts [32] and, surprisingly, can be modulated through the choice of activator used for the phosphoramidite coupling step to modestly bias the stereochemistry at the PS linkages toward the *R*p configuration. In addition, we previously showed that Lin28B, a prominent oncoprotein, is a key factor for the maintenance of cancer stem cells and a main driver in the development of prostate cancer [33]. We therefore first investigated the *in vitro* and *in vivo* activity of a PS siRNA directed against Lin28B after complexation with Lipofectamine or *in vivo* jetPEI, respectively. We then assessed the metabolic stability of the PS siRNA in different biological environments and compared it to that of current state-of-the-art chemically-modified siRNAs. We found that this simplistic modification pattern provided a substantial increase in metabolic stability compared to the classical PO siRNA without significantly compromising its silencing activity. Moreover, the nuclease stability of the PS siRNA matched the stability of nanoparticle-free patisiran in mouse serum and surpassed it in rat liver tritosome extracts, but did not reach the nuclease resistance of the fully chemically stabilized siRNAs.

Finally, we conducted a label-free biodistribution study in mice and evaluated the antitumor effect of phosphate-buffered saline (PBS)-formulated PS siRNA in a xenograft model of aggressive prostate cancer. The PS siRNA was recovered from several tissues after a single subcutaneous (s.c.) injection of the PBS-formulated siRNA in mice, with liver and kidney being the main sites of accumulation. Although an antitumor effect in a prostate cancer xenograft model could be demonstrated after delivery of the jetPEI-formulated PS siRNA, no inhibition of tumor growth was observed after a short-term treatment with the free siRNA.

## Materials and Methods

### Oligonucleotide synthesis

Chemicals for oligonucleotide synthesis were purchased from Sigma Aldrich (Steinheim, Germany), Fluorochem (Hadfield, United Kingdom), and TCI (Eschborn, Germany). Phosphoramidites were obtained from Thermo Fisher Scientific (Waltham, MA). Oligonucleotides were synthesized on a MM12 synthesizer from Bio Automation (BioAutomation Corp., Irving, TX) using 8 mL synthesis columns (BioAutomation), 300 mg of 500 Å UnyLinker CPG (ChemGenes, Wilmington, MA), and standard 2′-*O*-tert-butyldimethylsilyl (2′-*O*-TBDMS), 2′-OMe (Thermo Fisher Scientific), or 2′-fluoro-2′-deoxy phosphoramidites (ChemGenes). Phosphoramidites were prepared as 0.08 M solutions in dry acetonitrile (ACN); the activator 5-(Benzylthio)-1H-tetrazole (Biosolve BV, Valkenswaard, Netherlands) was prepared as a 0.24 M solution in dry ACN. Coupling time was 300 s. Oxidizer was prepared as a 0.02 M I_2_ solution in tetrahydrofuran (THF)/pyridine/H_2_O (70:20:10, v/v/v); sulfurization was carried out using a 0.1 M solution of ((dimethylamino-methylidene)amino)-3H-1,2,4-dithiazoline-3-thione (Glen Research, Sterling, VA) in dry pyridine/ACN (9:1) with a contact time of 600 s. Capping reagent A was THF/lutidine/acetic anhydride (8:1:1), and capping reagent B was 16% *N*-methylimidazole/THF. Detritylations were performed using 3% dichloroacetic acid in dichloromethane.

Oligonucleotides were cleaved from the solid support, and the protecting groups on the exocyclic amino groups and the backbone were removed using a 1:1 mixture of 40% aqueous methylamine and 25% aqueous ammonia for 1 h at 65°C. 2′-*O*-TBDMS groups were removed using a freshly-prepared mixture of *N*-methyl-2-pyrrolidone (120 μL), triethylamine (TEA; 60 μL), and TEA.3HF, (80 μL) at 70°C for 2 h. Desilylation was quenched with trimethylethoxysilane (400 μL); then diisopropyl ether (200 μL) was added to precipitate the oligonucleotide. The precipitate was dissolved in H_2_O and purified whilst still 4,4′-dimethoxytrityl (DMT)-protected (DMT-on) on an Agilent 1200 series high performance liquid chromatography (HPLC) fitted with a Waters XBridge Oligonucleotide BEH C18 column (10 × 50 mm, 2.5 μm) at 65°C. Fractions were pooled, dried in a SpeedVac, and treated for 15 min with 40% acetic acid at room temperature.

After drying in a SpeedVac, oligonucleotides were dissolved in H_2_O and subjected to a second purification on reversed-phase HPLC. Gradient for DMT-on purification: 10%–50% eluent B in 5 min, flow rate = 5 mL/min. DMT-off purification: 2%–20% eluent B in 8 min, flow rate = 5 mL/min. Eluent A was 0.1 M triethylammonium acetate, pH 8.0. Eluent B was ACN. The integrities of purified oligonucleotides were confirmed by liquid chromatography–mass spectrometry analysis on an Agilent 1200/6130 system fitted with a Waters acquity UPLC OST C-18 column (2.1 × 50 mm, 1.7 μm) at 65°C, with a gradient of 5%–35% eluent B in 14 min with a flowrate of 0.3 mL/min. Eluent A was aqueous hexafluoroisopropanol (0.4 M) containing TEA (15 mM). Eluent B was methanol.

### Nuclease stability assays

siRNAs (11.5 μM) were incubated in 50% mouse serum (S-25M; Sigma Aldrich) or rat liver tritosome extracts (Xenotech LLC Kansas City, KS) for the indicated time points and analyzed by ion-exchange HPLC. UV-absorption was monitored at 260 nm. Peak areas were normalized to the respective 0 min time point. Rat liver tritosomes were standardized to 0.5 U/mL acid phosphatase in 20 mM citrate buffer (pH = 5) as previously described [15,34]. Incubation was quenched through digestion in MasterPure tissue lysis solution (Epicentre, WI) containing 0.8 mg/mL proteinase K (Roche) at 65°C for 30 min and stored at −80°C until analysis. Shortly before analysis, sodium dodecyl sulfate (SDS) was precipitated through addition of 3 M KCl to a final concentration of 0.6 M and centrifugation at 4°C, 12*g* for 10 min. Cleared supernatants were analyzed on a Hitachi VWR LaChrom Elite HPLC fitted with a DNA Pac PA200 (4 × 250 mm) anion exchange column and a DNA Pac PA200 (4 × 50 mm) guard column at 30°C. The gradient was 100% A for 2 min, followed by 54% eluent B within 5 min, increase to 100% B within 2 min, hold 100% B for 1 min, switch to 100% A within 2 min, and hold 100% A for 5 min. Eluent A was a 1:1 mixture (v:v) of buffer A and ACN. Buffer A was an aqueous solution of 1 mM ethylenediaminetetraaceticacid (EDTA) and 25 mM Tris HCl (pH = 8.5). Eluent B was a 1:1 mixture (v:v) of buffer B and ACN. Buffer B was an aqueous solution of 1 mM EDTA, 25 mM Tris HCl, and 1.6 M NaClO_4_ (pH = 8.5).

### Peptide nucleic acid hybridization assay

Peptide nucleic acid (PNA) hybridization assays were performed based on literature protocols [35–38]. Tissues were pulverized in liquid nitrogen and homogenized in 1 mL MasterPure tissue lysis solution (Epicentre) supplemented with 0.8 mg/mL Proteinase K (Roche) in a tissue lyser (Qiagen, Germany) using Qiagen stainless steel beads (5 mm) for 6 min with a frequency of 30 Hz. Tissue homogenates were centrifuged at 10*g*, 4°C for 5 min, and supernatant was transferred to clean Eppendorf tubes. Then, proteinase K digestion was performed at 65°C for 30 min. SDS was precipitated as described for nuclease stability assays. To 100 μL of cleared lysate was added 95 μL hybridization buffer and 5 μL atto425-PNA probe (8 μM in 20% ACN; Panagene, Korea). Hybridization buffer was an aqueous solution of 50 mM Tris (pH = 8.8) containing 10% ACN.

PNA hybridization was performed on a thermocycler according to the following temperature program: 95°C for 15 min followed by 20°C for 15 min. After hybridization, supernatants were pooled and centrifuged again (10*g*, 4°C for 15 min). Cleared supernatants were subjected to anion exchange HPLC as described for nuclease stability assays, but with a column temperature of 50°C, and fluorescence at 484 nm (excitation at 436 nm) was monitored on a Hitachi VWR LaChrom Elite L-2485 detector.

### Cell culture and transfections

HEK293T cells were maintained in Dulbecco's modified Eagle's medium GlutaMAX (31966, Gibco^®^; Life Technologies) supplemented with 10% of fetal bovine serum. Transfections were performed according to the manufacturer's protocol with Lipofectamine 2000 (11668027; Thermo Fisher Scientific). Control siRNAs are listed in Supplementary Table S1. Experimental details for western blotting and the albumin binding assay are described in the Supplementary Methods.

### SYBR Green reverse transcription–quantitative polymerase chain reaction

Total RNA was extracted using TRIzol (15596026; Invitrogen) according to the manufacturer's protocol. RNA concentrations were measured on a NanoDrop 2000 spectrophotometer. For mRNA analysis, 1 μg of total RNA was reverse transcribed using the High Capacity cDNA Reverse Transcription Kit (4368814; Applied Biosystems) according to the manufacturer's protocol. Reverse transcription was performed on a thermocycler S1000 (Bio-Rad) according to the following program: 25°C, 10 min; 37°C, 120 min; and 85°C, 5min.

SYBR Green quantitative polymerase chain reaction was performed on a LightCycler 480 instrument (Roche) using KAPA SYBR FAST for Roche LightCycler 480 (KK4610; Sigma-Aldrich). PCR primers were designed using the Universal ProbeLibrary System Assay Design (Roche) and were ordered from Microsynth (Balgach, Switzerland). Primer sequences (5′-3′) were: Lin28B GAAAAGAAAACCAAAGGGAGATAGA (forward), GAGGTAGACTACATTCCTTAGCATGA (reverse); GAPDH AGCCACATCGCTCAGACAC (forward), GCCCAATACGACCAAATCC (reverse); and ACTB CCAACCGCGAGAAGATGA (forward), CCAGAGGCGTACAGGGATAG (reverse). Cycling conditions were 95°C, 10 min and 40 cycles of 95°C, 15 s; 60°C, 1 min. Each reaction was carried out in three technical replicates. Cp values were extracted with the LightCycler software V1.5 (Roche).

### Animal experiments

All procedures involving animals were conducted in conformity with the institutional guidelines for animal experimentation of the Institute of Oncology Research Bellinzona and in compliance with national and international policies. Study protocols were approved by the Swiss Veterinary Authority. Athymic nude mice were purchased from Charles River and the Harlan Laboratories and maintained under pathogen-free conditions with food and water *ad libitum*. The general health status of all animals was monitored daily. For the biodistribution study, five athymic nude mice (Balb/c nu/nu, 4–6 weeks old; Charles River laboratories) received a single s.c. injection of 50 mg/kg siRNA II in PBS. As a control, two mice received a single s.c. injection of PBS alone. Mice were sacrificed 4 h after the injection, and the following tissues were collected: liver, kidney, spleen, lung, heart, skeletal muscle, prostate, and blood.

For the tumor xenograft study using *in vivo* jetPEI formulated siRNA II (DU145 xenografts), mice were purchased from the Harlan Laboratories. DU145 cells were mixed with Matrigel and injected (5 × 10^6^ cells/site) in the flank of athymic nude mice (Balb/c nu/nu, 4–6 weeks old; *n* = 4/group). Mice with s.c. tumor xenografts (ca. 100 mm^3^) received intraperitoneal injections of siCon III, siRNA I, or siRNA II, each formulated with *in vivo* jetPEI (Polyplus Transfection) at the dose of 2 mg/kg thrice a week (Monday, Wednesday, Friday) for 3 weeks. For the xenograft study using PBS-formulated siRNA II, each cohort consisted of seven mice (PC3 xenografts). PC3 (3 × 10^6^ cells/site) cells were inoculated with Matrigel in the flank of NSG mice (Charles River laboratories), and treatment was started when tumors were ca. 100 mm^3^. Mice received three s.c. injections (Monday, Wednesday, Friday) of siRNA II (50 mg/kg, in PBS), siCon II (50 mg/kg, in PBS), or siRNA I (5 mg/kg, formulated with *in vivo* jetPEI) per week for 2 weeks. Tumor growth in both studies was monitored twice a week by caliper. Tumor volume and weight were measured at the end of the treatment.

## Results

**FIG. 1.**
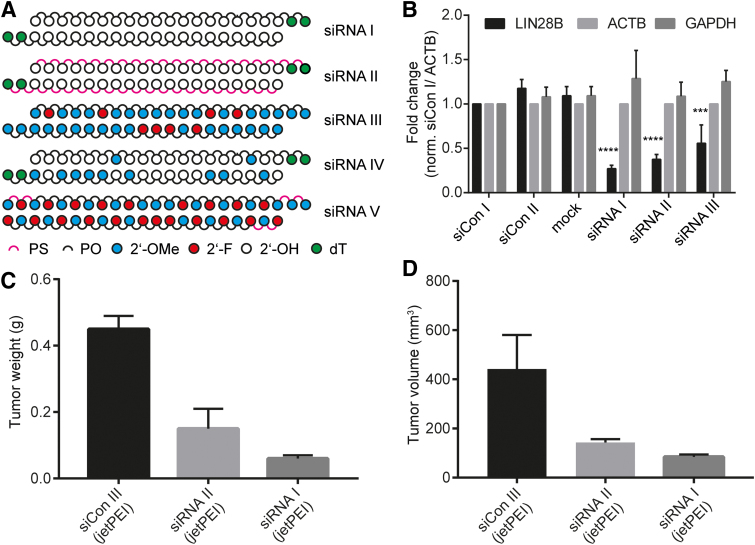
*In vitro* and *in vivo* silencing activity of siRNAs in this study. **(A)** Chemical architecture of siRNA variants investigated. **(B)** Reverse transcription–quantitative polymerase chain reaction of Lin28B, ACTB, and GAPDH after transfection of 40 nM siRNAs I–III (Lipofectamine 2000) in HEK 293T cells. Results normalized to ACTB and siCon I. siCon I and II are scrambled siRNA I and II, respectively. *Asterisks* indicate statistical significance to mock treatment calculated by one-way analysis of variance and Dunnett's *post hoc* test. ***P < 0.001; ****P < 0.0001, *n* = 3. **(C)** Tumor weight and **(D)** tumor volume at the end of treatment. DU145 prostate cancer cells (5 × 10^6^ cells/injection site) were implanted subcutaneously in athymic nude mice, and treatment started when tumors were 100 mm^3^. Mice (*n* = 4/group) received intraperitoneal injections of siCon III, siRNA I, or siRNA II (three injections/week for 3 weeks) at a dose of 2 mg/kg, formulated with *in vivo* jetPEI [data from **(C)**], and **(D)** was previously published in part (siRNA I) [32] and is included here for comparison. siRNA, small interfering RNA. Color images are available online.

We synthesized three siRNA constructs directed against Lin28B mRNA (Fig. 1A); namely, an unmodified siRNA encompassing a classical two nt dT overhang (siRNA I), a PS analog thereof (siRNA II), and a fully 2′-OMe, 2′-F stabilized variant (siRNA III) that was modified according to recently published design guidelines [39]. We have previously described the *in vitro* and *in vivo* targeting properties of siRNA sequences siRNA I and siRNA II [32,33,40,41]. We also prepared two reference siRNAs, corresponding to the recently approved patisiran [12,13] without its LNP formulation (siRNA IV) and the currently clinically investigated fitusiran [42,43] without the GalNAc group (siRNA V) (Table 1 and Supplementary Fig. S1).

**Table 1.** Sequences of Small Interfering RNAs

siRNA	Strand	Sequence (5′-3′)	M calc	M found	Target
siRNA I	AS	AAAUCCUUCCAUGAAUAGUTT	6599.07	6598.83	Lin28B
	S	ACUAUUCAUGGAAGGAUUUTT	6656.09	6655.29	
siRNA II	AS	**AAAUCCUUCCAUGAAUAGUTT**	6920.38	6919.45	Lin28B
	S	**ACUAUUCAUGGAAGGAUUUTT**	6977.40	6976.11	
siRNA III	AS	**aA**aucCuuccaugAaUagu**uu**	6913.65	6912.87	Lin28B
	S	**ac**uauuCaUGGaaggauuu**uu**	6970.65	6969.91	
siRNA IV	AS	AUGGAAuACUCUUGGUuACTT	6660.07	6659.25	TTR
	S	GuAAccAAGAGuAuuccAuTT	6764.32	6763.61	
siRNA V	AS	**uU**gAaGuAaAuggUgUuAaCc**ag**	7675.03	7674.09	Serpinc 1
	S	**Gg**UuAaCaCCAuUuAcUuCaA	6784.26	6783.30	

*Upper case* is RNA, *upper case* underlined is 2′-F, *lower case* is 2′-OMe, PS linkages are in *bold*. Sequences of siRNA IV and V retrieved from patents US20170307608A1 and US20170159053A1, respectively.

siRNA, small interfering RNA; PS, phosphorothioate.

Transfection of siRNAs I-III into HEK293T cells that express detectable levels of Lin28B revealed that siRNA II was marginally less active than the unmodified siRNA I (Fig. 1B and Supplementary Fig. S2). Surprisingly, the loss in silencing activity in the HEK293T cells was more pronounced for siRNA III (Fig. 1B), possibly due to the change of the aforementioned asymmetric modification pattern to a classical siRNA format and the presence of 2′-OMe nucleosides in positions 9/10 (antisense strand), which are often modified with 2′-F as in vutrisiran [44] or fitusiran (Table 1). An alternative explanation could be a poor intracellular phosphorylation efficiency of the modified RNA [34,45]. In a previous investigation, we described how jetPEI-formulated siRNA I inhibits tumor growth in a mouse DU145-xenograft model [33]. When the fully PS-modified siRNA II was tested under the same conditions, it showed a modestly reduced level of inhibition of tumor growth compared to siRNA I (Fig. 1C, D) [33]. We therefore questioned whether the full PS backbone might provide sufficient stabilization and suitable pharmacokinetic properties to enable *in vivo* activity also in the absence of a delivery vehicle.

**FIG. 2.**
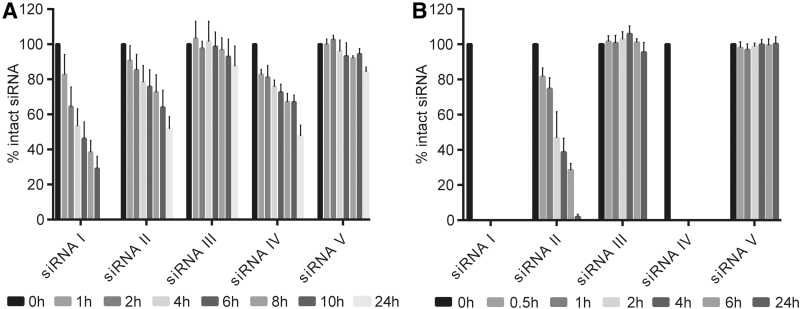
Nuclease stability in different biological matrices. Stability of siRNAs I–V in **(A)** 50% murine serum and **(B)** rat liver tritosome extracts. siRNAs were incubated either in 50% murine serum or standardized rat liver tritosome extracts (pH = 5), and aliquots were analyzed by ion-exchange high performance liquid chromatography at the indicated time points (*n* = 3).

Upon s.c. injection, siRNAs are exposed to a diverse set of nucleases during circulation, extravasation, and cell entry. Although our understanding of the cellular uptake of therapeutic oligonucleotides is limited, siRNAs are thought to be transported in endosomal vesicles that are subsequently fused with lysosomes [46–48]. While several nanoparticle or polymer based formulations have been developed to facilitate endosomal escape [49], nuclease stability during endosomal trafficking is required for the administration of oligonucleotides formulated in PBS and has been identified as a key determinant of the *in vivo* efficacy of GalNAc-conjugated siRNAs [15]. Rat liver tritosomes are an *in vitro* surrogate for cellular lysosomal compartments [50]. We therefore investigated the nuclease resistance of siRNAs I–V in two different biological matrices: mouse serum (Fig. 2A and Supplementary Fig. S3) and rat liver tritosome extracts (Fig. 2B and Supplementary Fig. S4). As expected, unmodified siRNA I was largely degraded within 24 h in mouse serum and within 30 min in tritosome extracts, whereas fully-modified siRNAs III and V showed superior stability. In contrast, the nuclease stability of siRNA II was markedly increased compared to that of siRNAs I and IV. Particularly the stark difference between siRNAs II and IV in the tritosome assay, despite similar serum stability profiles, encouraged us to further investigate siRNA II as an alternative scaffold for nanoparticle-free delivery. In addition, an electrophoretic mobility shift assay based albumin binding study suggested a moderately increased albumin binding of siRNA II compared to siRNA I (Supplementary Fig. S5). The albumin binding appears to be transient as indicated by an albumin-dependent smear instead of an increase of the slow migrating band and might contribute to a more favorable pharmacokinetic profile.

**FIG. 3.**
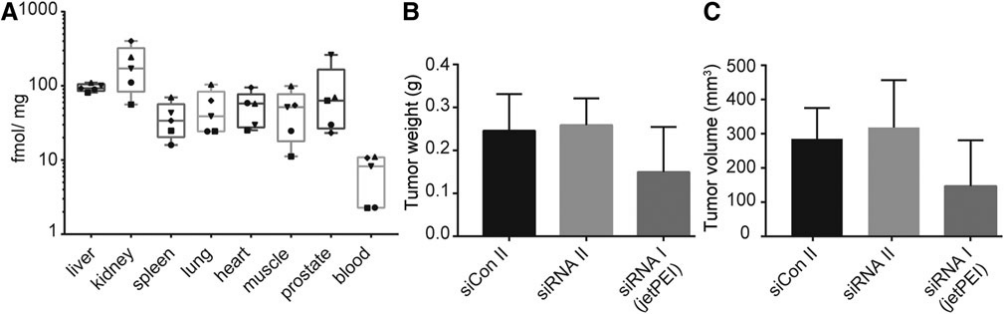
Biodistribution and *in vivo* silencing activity of phosphorothioate siRNA II. **(A)** Biodistribution profile of siRNA II. Nude mice received a single subcutaneous injection of 50 mg/kg. Mice were sacrificed 4 h after the injection. Quantification *via* PNA hybridization assay. *Symbols* indicate individual mice. Median is indicated by a *horizontal mark*. The *edges* of each *box* mark the 25^th^ and 75^th^ percentile. *Whiskers* extend to lowest and highest values. One femtomole per milligram tissue corresponds to 0.014 ng/mg. **(B)** Tumor weight and **(C)** tumor volume after repeated injections of siRNA II (50 mg/kg in PBS), siCon II (50 mg/kg in PBS), or siRNA I (5 mg/kg, *in vivo* jetPEI) in PC3 xenograft bearing mice. Mice received three injections per week for 2 weeks, *n* = 7. NB: The response of the PC3-xenografts toward jetPEI formulated siRNA I was weaker than for DU145 xenografts (Fig. 1C, D). PBS, phosphate-buffered saline; PNA, peptide nucleic acid hybridization assay.

Hence, we conducted a label-free biodistribution study for which nude mice received either a single s.c. injection of 50 mg/kg siRNA II or PBS (Fig. 3A and Supplementary Fig. S6, S7). A range of tissues were harvested 4 h after the injection and analyzed by a PNA hybridization assay [35–38]. Main sites of accumulation were liver and kidney, in line with previous reports of naked administration of chemically modified siRNAs [18,26,31,51]. Lowest levels were found in blood, consistent with a fast excretion and tissue distribution of circulating siRNAs. Total amounts of siRNA in tissues were 10 × lower compared to a previous study that used Dynamic Polyconjugate™ formulation [35] and ∼10–100 × lower compared to GalNAc- or lipid-conjugated fully modified siRNAs [15,31]. As amounts of siRNAs in the cytosol are generally much lower than the total tissue deposition [15], we wondered if the observed tissue accumulation led to productive cellular uptake [52]. Previous reports have indicated that PS-modified oligonucleotides can stimulate the uptake of co-delivered siRNAs in trans [53]. We therefore hypothesized that the full PS backbone chemistry of siRNA II might also facilitate cellular uptake in a comparable manner. To investigate this, we conducted a mouse xenograft study in which each animal received six injections of either PBS-formulated siRNA II or siCon II. *In vivo* jetPEI formulated siRNA I served as a positive control, and tumor growth was monitored over a period of 2 weeks. Although tumor growth was generally slower than expected, no reduction in tumor growth was observed after administration of PBS-formulated siRNA II compared to the corresponding scrambled control (siCon II) (Fig. 3B, C), whereas some activity was observed after administration of siRNA I. The animal weight at the end of treatment was not significantly different between the groups (Supplementary Fig. S8B).

We therefore concluded that either the metabolic stability (Fig. 2A, B) or protein binding abilities (Supplementary Fig. S5) of siRNA II did not produce sufficient tissue accumulation or that the double-stranded format did not stimulate productive cellular uptake in a comparable manner as has been described for single-stranded PS oligonucleotides. Nevertheless, as particularly the use of 2′-F building blocks has recently been questioned due to a potential risk of genotoxicity [54], further research will focus on other alternative strategies that allow the construction of siRNAs for systemic delivery without the need for extensive chemical modification.

## Discussion

To be efficient in patients, state-of-the-art siRNAs are constructed from three essential components: a fully modified RNA core that conveys metabolic stability, a targeting moiety which mediates uptake into the desired cell population, and a linker which provides optimal spacing for interaction with the designated cell surface receptors. However, the increasing complexity of the siRNA core poses a challenge in terms of risk assessment of secondary effects originating from the long-term exposure to chemically modified RNA metabolites. In particular the use of 2′-F building blocks has recently come under scrutiny because of the potential incorporation of modified nucleosides into host DNA and RNA [44,54].

In this study we show that uniform PS modification of a siRNA directed against the oncoprotein Lin28B leads to a substantial increase in metabolic stability compared to a classical PO siRNA, without greatly compromising the gene silencing activity. We found that the nuclease stability of PS siRNAs was not only increased compared to the PO variant but also matched the stability of nanoparticle-free patisiran in mouse serum and surpassed both in rat liver tritosome extracts. However, it did not reach the stability of a fully chemically stabilized siLin28B analog or that of the fitusiran siRNA core structure. A label-free biodistribution study revealed liver and kidney as the main sites of tissue accumulation after s.c. administration of the PS siRNA formulated in PBS. However, no inhibition of tumor growth was observed after naked delivery of the PS siRNA in a PC3 xenograft study. Possible reasons could be a low penetration and uptake of the siRNA into the tumor tissue or that the observed level of metabolic stability was not sufficient to support the treatment regimen but would require a more frequent dosing. To gain further insights into the ability of siRNAs to penetrate into tumor tissues, it will be of value to investigate the uptake into different sections of the tumor in an orthotopic model.

Despite the lack of a delivery agent-free antitumor effect, we anticipate our study to be a starting point to develop alternative siRNA scaffolds that can be degraded into naturally occurring metabolites to minimize the risk of genotoxicity after long-term exposure to non-natural RNA metabolites. For example, the insertion of PS linkages of defined stereochemistry in hotspot regions for nuclease degradation offers a possibility to modulate the pharmacokinetic profile without the formation of additional metabolites. Recent developments regarding the use of heavily-modified siRNAs have led to a reduction of the 2′-F content in favor of 2′-OMe nucleosides, which also limits patient exposure [39,44]. Furthermore, Lin28B is a promising target in different cancers, and the development of new siLin28B analogs, possibly in conjunction with novel targeting units, holds great potential for future antitumor therapies.

## Supplementary Material

Supplemental data

Supplemental data

Supplemental data

Supplemental data

Supplemental data

Supplemental data

Supplemental data
