# Supplementary material for: Pharmacodynamic and Pharmacokinetic Properties of Full Phosphorothioate Small Interfering RNAs for Gene Silencing In Vivo
Source: Nucleic Acid Ther. 2021 Jun 4;31(3):237–44. doi: 10.1089/nat.2020.0852 (PMC8215415; doi:10.1089/nat.2020.0852)
Supplement: Supplemental data [file Supp_FigS7-8-T1.pdf]

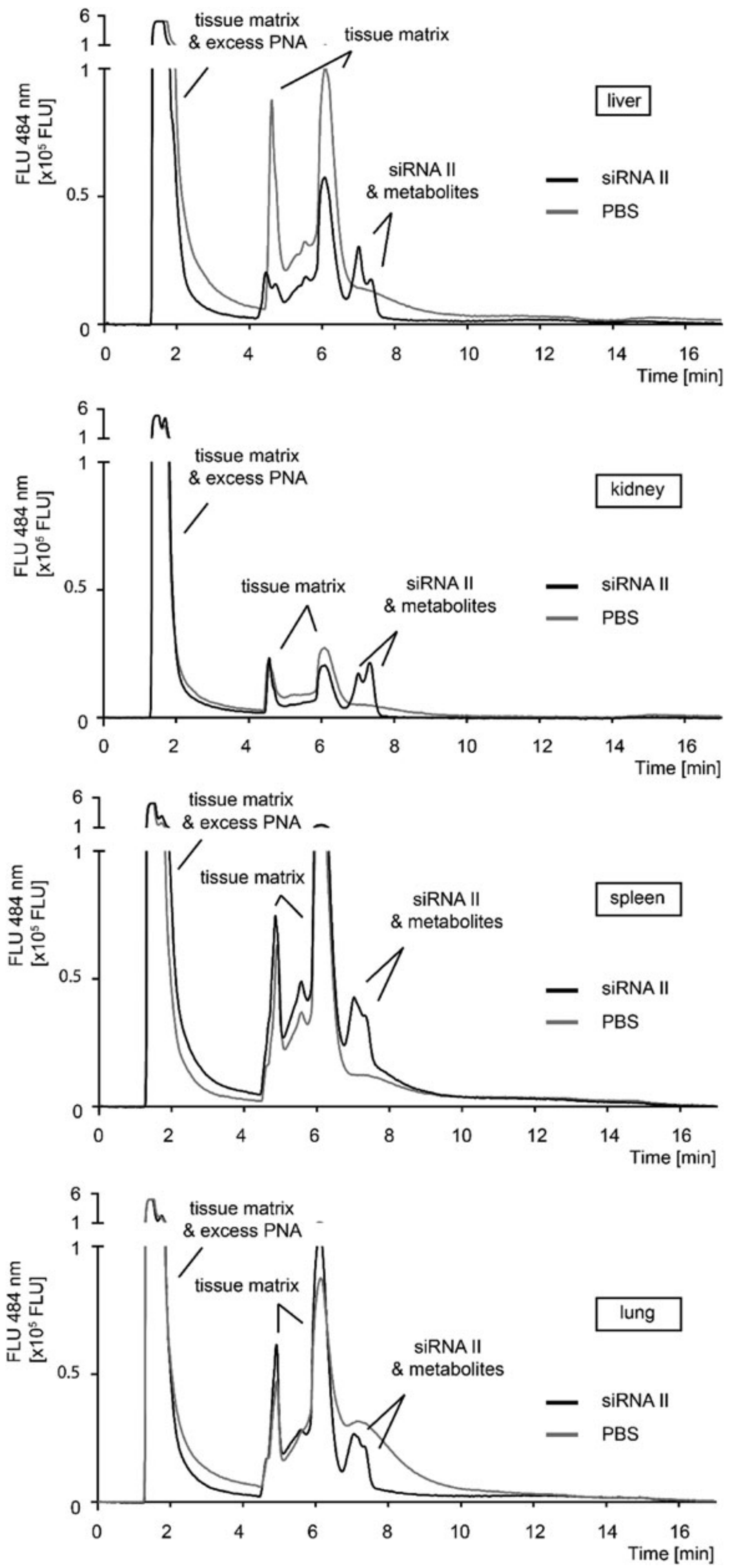

SUPPLEMENTARY FIG. S7. Chromatograms of anion-exchange HPLC analysis (fluorescence trace) from biodistribution study. Overlays of tissues from a mouse that was injected with 50 mg/kg siRNA II and a mouse that received PBS treatment are shown. Analysis was performed on a Hitachi VWR LaChrom Elite HPLC fitted with a DNA Pac PA200 (4×250 mm) anion exchange column and a DNA Pac PA200 (4×50 mm) guard column at 50°C. The gradient was 100% A for 2 min, followed by 54% eluent B within 5 min, increase to 100% B within 2 min, hold 100% B for 1 min, switch to 100% A within 2 min, and hold 100% A for 5 min. Eluent A was a 1:1 mixture (v:v) of buffer A and ACN. Buffer A was an aqueous solution of 1 mM EDTA and 25 mM Tris HCl (pH = 8.5). Eluent B was a 1:1 mixture (v:v) of buffer B and ACN. Buffer B was an aqueous solution of 1 mM EDTA, 25 mM Tris HCl, and 1.6 M $NaClO_4$ (pH = 8.5). Note: We have no explanation for the high background levels of material in the PBS sample from the lung. The chromatogram from the prostate sample shown here corresponds to the mouse with the highest siRNA accumulation in the prostate. PBS, phosphate-buffered saline.

(*continued*)

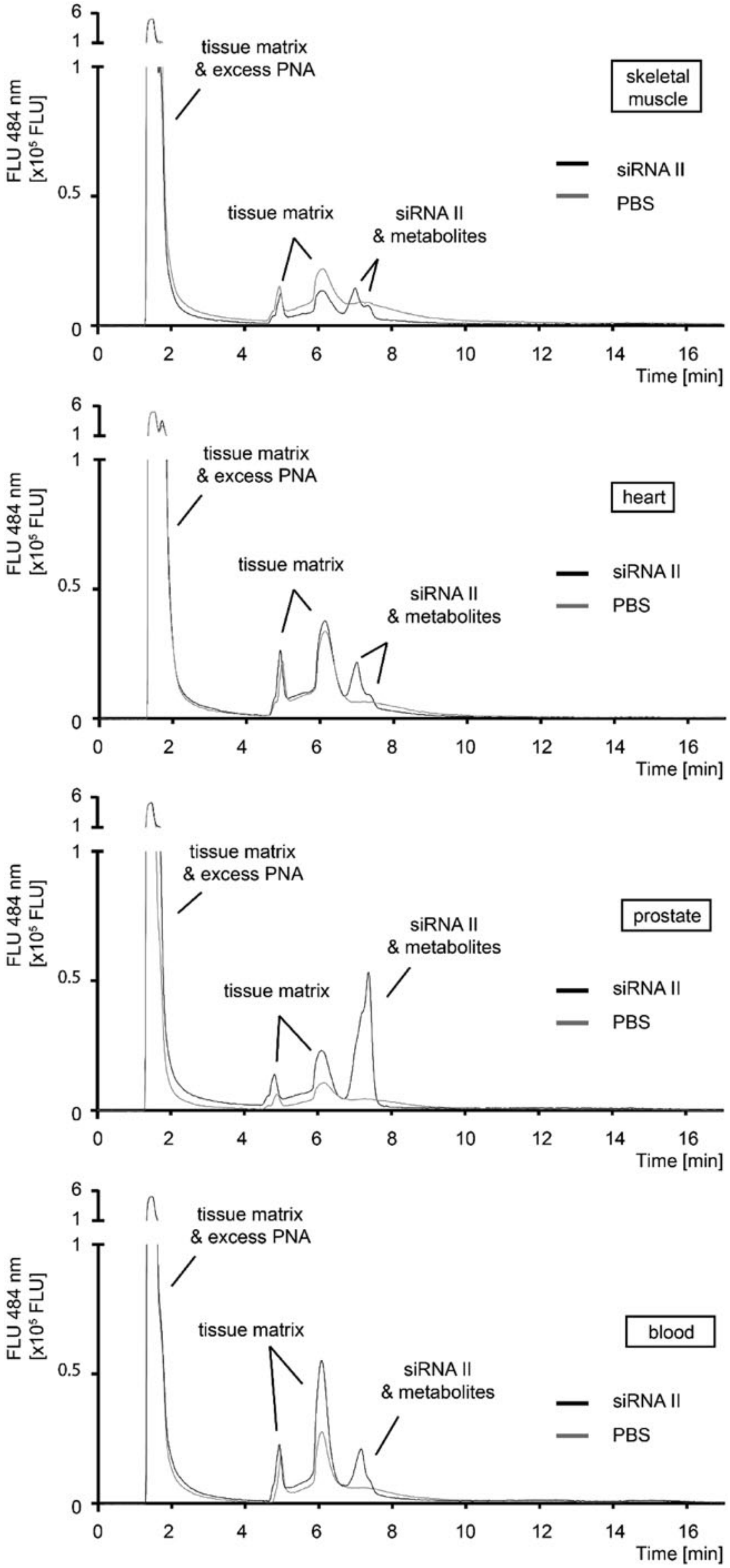

SUPPLEMENTARY FIG. S7. (*Continued*).

(*continued*)

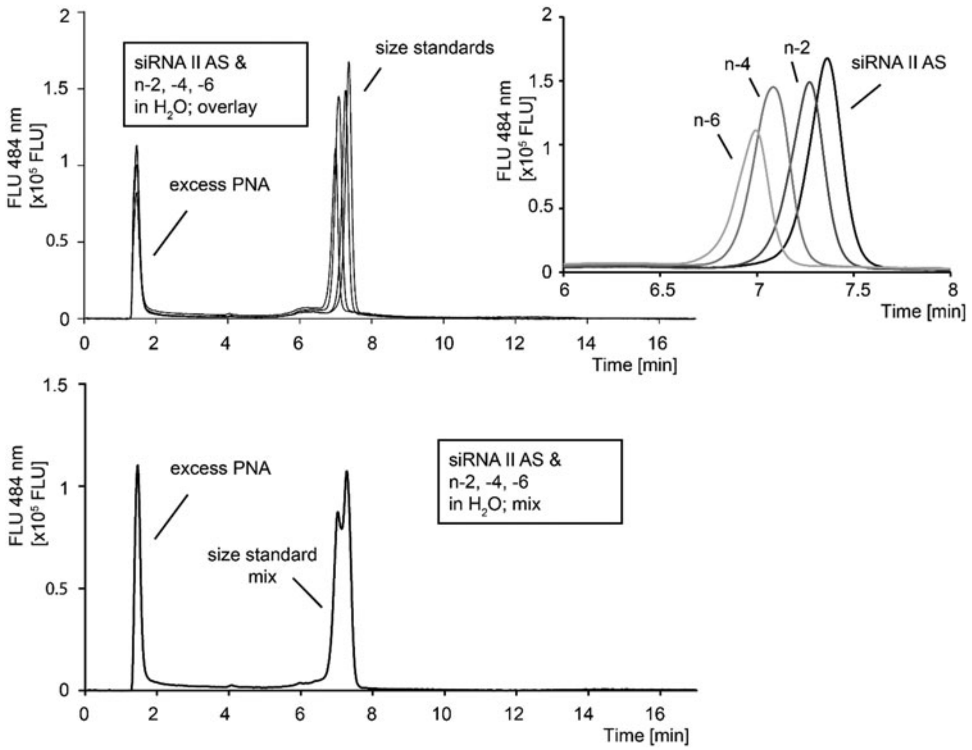

SUPPLEMENTARY FIG. S7. (*Continued*).

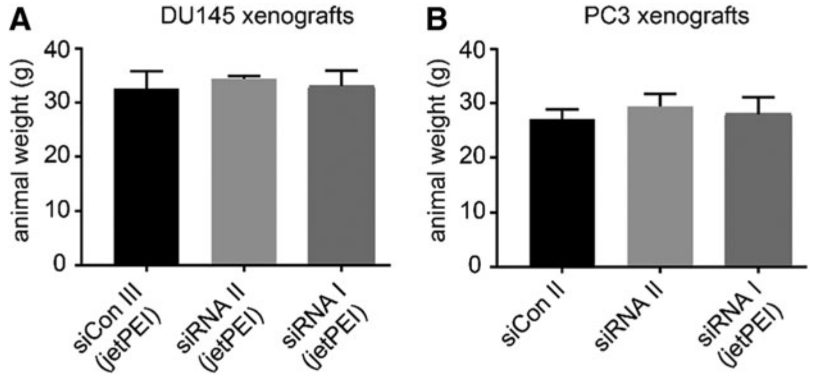

SUPPLEMENTARY FIG. S8. Animal weight at the end of treatments. **(A)** Animal weight at the end of the treatment of DU145 xenografts with *in vivo* jetPEI formulated siRNAs (Fig. 1C, D). DU145 prostate cancer cells (5×10^6 cells/injection site) were implanted subcutaneously in athymic nude mice and treatment started when tumors were 100 mm^3. Mice ($n = 4$/group) received intraperitoneal injections of siCon III, siRNA I, or siRNA II (three injections/week for 3 weeks) at a dose of 2 mg/kg, formulated with *in vivo* jetPEI. **(B)** Animal weight at the end of the treatment of PC3 xenografts with siRNA II (50 mg/kg in PBS), siCon II (50 mg/kg in PBS), or siRNA I (5 mg/kg, *in vivo* jetPEI). PC3 prostate cancer cells (3×10^6 cells/injection site) were implanted subcutaneously in athymic nude mice, and treatment started when tumors were 100 mm^3. Mice received 3 injections per week for 2 weeks, $n = 7$ (Fig. 3B, C).

Supplementary Table S1. Sequences of Control Small Interfering RNAs

siRNA	*Strand*	*Sequence (5′-3′)*	*M calc*	*M found*
siCon I	AS	AUAAGUACGUUCACUACUATT	6599.07	6602.12
	S	UAGUAGUGAACGUACUUAUTT	6656.09	6659.10
siCon II	AS	**AUAAGUACGUUCACUACUATT**	6920.38	6918.60
	S	**UAGUAGUGAACGUACUUAUTT**	6977.40	6976.12

Upper case is RNA; PS linkages are in *bold*. siCon III was siGL3 [3] and was purchased from Ambion.
siRNA, small interfering RNA; PS, phosphorothioate.
