# Supplementary material for: Pharmacodynamic and Pharmacokinetic Properties of Full Phosphorothioate Small Interfering RNAs for Gene Silencing In Vivo
Source: Nucleic Acid Ther. 2021 Jun 4;31(3):237–44. doi: 10.1089/nat.2020.0852 (PMC8215415; doi:10.1089/nat.2020.0852)
Supplement: Supplemental data [file Supp_DataS1.pdf]

Supplementary Data

Supplementary Methods

Western blotting

A total of 40,000 HEK 293T cells/well were seeded in a 24-well plate. Transfection of small interfering RNAs (siRNAs) at a final concentration of 40 nM using Lipofectamine 2000 (Thermo Fisher) was performed 12 h after seeding according to the manufacturer's instructions. Mock treated cells received the Lipofectamine 2000 mix without siRNAs. Cells were harvested 48 h post-transfection using RIPA lysis buffer (Sigma). Protein concentrations were determined using a BCA assay (Thermo Fisher) according to the manufacturer's recommendations. Two micrograms of total protein was prepared in 1 × Laemmli buffer (Bio-Rad) and boiled at 95°C for 5 min, followed by polyacrylamide gel electrophoresis (PAGE) separation on a 4%–20% sodium dodecyl sulfate (SDS) Mini-PROTEAN TGX Precast Gel (Bio-Rad) and wet-blotting on a polyvinylidene difluoride membrane (GE Healthcare). The membrane was blocked for 1 h at room temperature with 5% milk in phosphate-buffered saline (PBS) containing 0.05% Tween 20 (PBST). Overnight staining with Lin28B rabbit primary antibody (Cell Signaling) was performed at 4°C at a dilution ratio of 1:3,000 in PBST. After washing, the membrane was incubated with a peroxidase-labelled goat anti-rabbit secondary antibody (KPL) in a dilution of 1:10,000 in PBST for 90 min at ambient temperature. Chemiluminescence was induced through ECL prime western blotting detection (Amersham Biosciences) according to the manufacturer's protocol and visualized using a ChemiDoc device (Bio-Rad). The membrane was stripped for 15 min at 37°C with stripping buffer (Thermo Fisher), followed by washing, blocking, and overnight incubation with GAPDH antibody (mouse monoclonal antibody, OriGene) in a 1:10,000 dilution in PBST at 4°C. Then the membrane was washed and incubated with a 1:10,000 dilution of peroxidase-labelled goat anti-mouse secondary antibody (KPL) in PBST for 1 h at room temperature. Protein bands were quantified by densitometry using the analysis software ImageJ and normalized to GAPDH and mock treatment.

Albumin binding assay

A PAGE migration assay was used to evaluate siRNA binding to human serum albumin. The protocol was based on previous reports by Sarett *et al.* [1] and Hvam *et al.* [2]. An albumin stock solution was prepared by dissolving 2.73 mg human serum albumin (HSA, A3782; Sigma Aldrich) in 200 μL PBS (200 μM). Dilutions of 1:1 were prepared with PBS to obtain the following HSA concentrations: 200, 100, 50, and 25 μM. Of 20 μM siRNA stocks, 0.5 μL was added to 19.5 μL of the respective HSA solution or PBS. Final concentration of RNA was kept constant at 0.5 μM. Samples were incubated at 37°C for 2 h and then mixed with 6 × gel loading dye purple, no SDS (B7025S; New England Biolabs). Samples were analyzed on a 4%–20% precast native polyacrylamide gel (Mini-PROTEAN® TGX Stain-Free™ Protein Gel) using 1 × tris-borate-EDTA buffer (90 mM Tris, 90 mM boric acid, 2 mM ethylenediaminetetraaceticacid) at 60 V for 2 h. Ten microliters of sample was loaded per well. RNA was visualized with SYBR Gold Nucleic Acid stain (Invitrogen).
