# Supplementary material for: Pharmacodynamic and Pharmacokinetic Properties of Full Phosphorothioate Small Interfering RNAs for Gene Silencing In Vivo
Source: Nucleic Acid Ther. 2021 Jun 4;31(3):237–44. doi: 10.1089/nat.2020.0852 (PMC8215415; doi:10.1089/nat.2020.0852)
Supplement: Supplemental data [file Supp_FigS1.pdf]

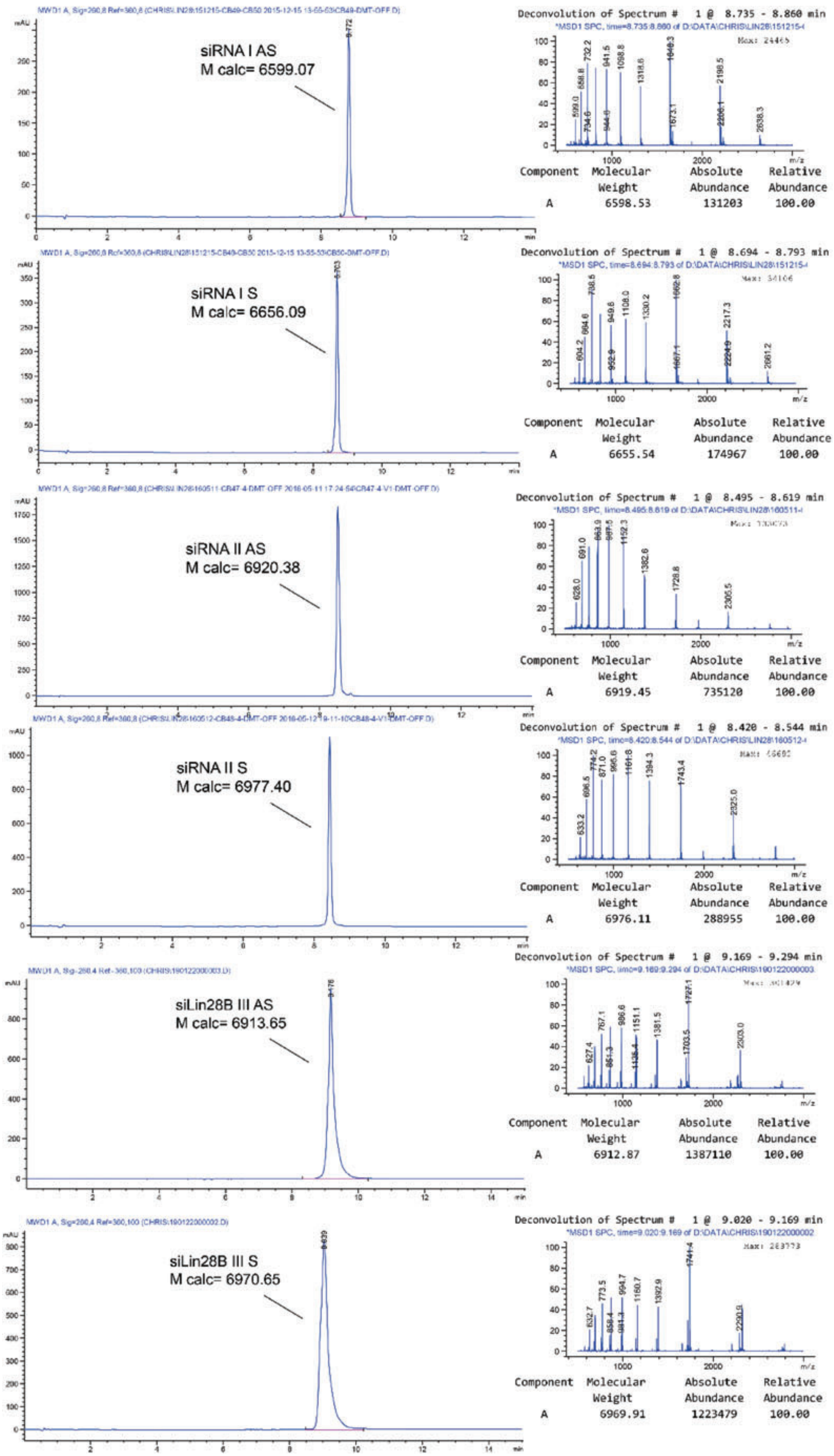

SUPPLEMENTARY FIG. S1. Liquid chromatography–mass spectrometry analysis of siRNAs I–V. Analysis was performed on an Agilent 1200/6130 system fitted with a Waters Acquity UPLC OST C-18 column (2.1×50 mm, 1.7 μm) at 65°C, with a gradient of 5%–35% eluent B in 14 min with a flow rate of 0.3 mL/min. Eluent A was aqueous hexafluoroisopropanol (0.4 M) containing triethylamine (15 mM). Eluent B was methanol. UV trace (260 nm) and mass spectra are shown. siRNA, small interfering RNA.

(*continued*)

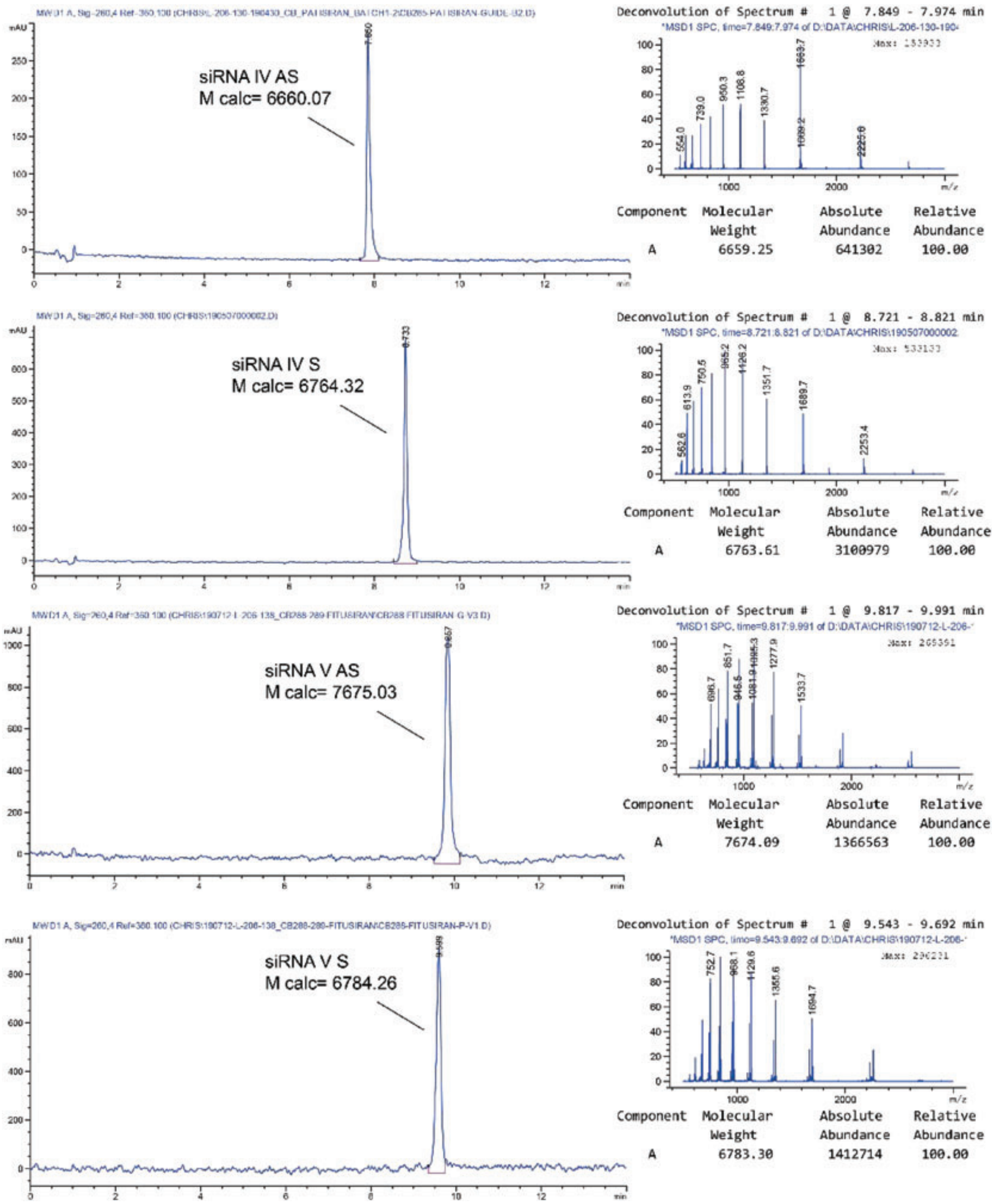

SUPPLEMENTARY FIG. S1. (*Continued*).
