# Supplementary material for: Pharmacodynamic and Pharmacokinetic Properties of Full Phosphorothioate Small Interfering RNAs for Gene Silencing In Vivo
Source: Nucleic Acid Ther. 2021 Jun 4;31(3):237–44. doi: 10.1089/nat.2020.0852 (PMC8215415; doi:10.1089/nat.2020.0852)
Supplement: Supplemental data [file Supp_FigS2.pdf]

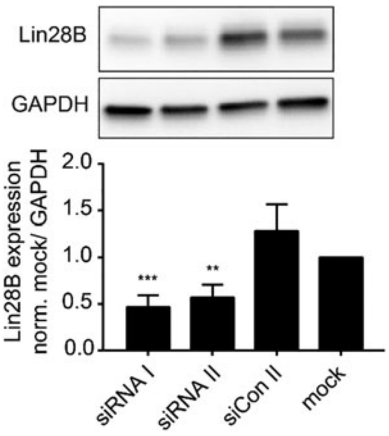

SUPPLEMENTARY FIG. S2. Activity of siRNA I and siRNA II in HEK 293T cells. Western blot analysis of Lin28B and GAPDH after transfection of 40 nM siRNAs (Lipofectamine 2000) in HEK 293T cells. Results normalized to GAPDH and mock treatment. Mean ± standard deviation of all experiments are the results of three independent replicates. Mock treatment was Lipofectamine 2000 alone. *Asterisks* indicate statistical significance to 0 nM treatment calculated by one-way analysis of variance and Dunnett's *post hoc* test. $^{**}P < 0.01$; $^{***}P < 0.001$.
