# Supplementary material for: Pharmacodynamic and Pharmacokinetic Properties of Full Phosphorothioate Small Interfering RNAs for Gene Silencing In Vivo
Source: Nucleic Acid Ther. 2021 Jun 4;31(3):237–44. doi: 10.1089/nat.2020.0852 (PMC8215415; doi:10.1089/nat.2020.0852)
Supplement: Supplemental data [file Supp_FigS4-S5.pdf]

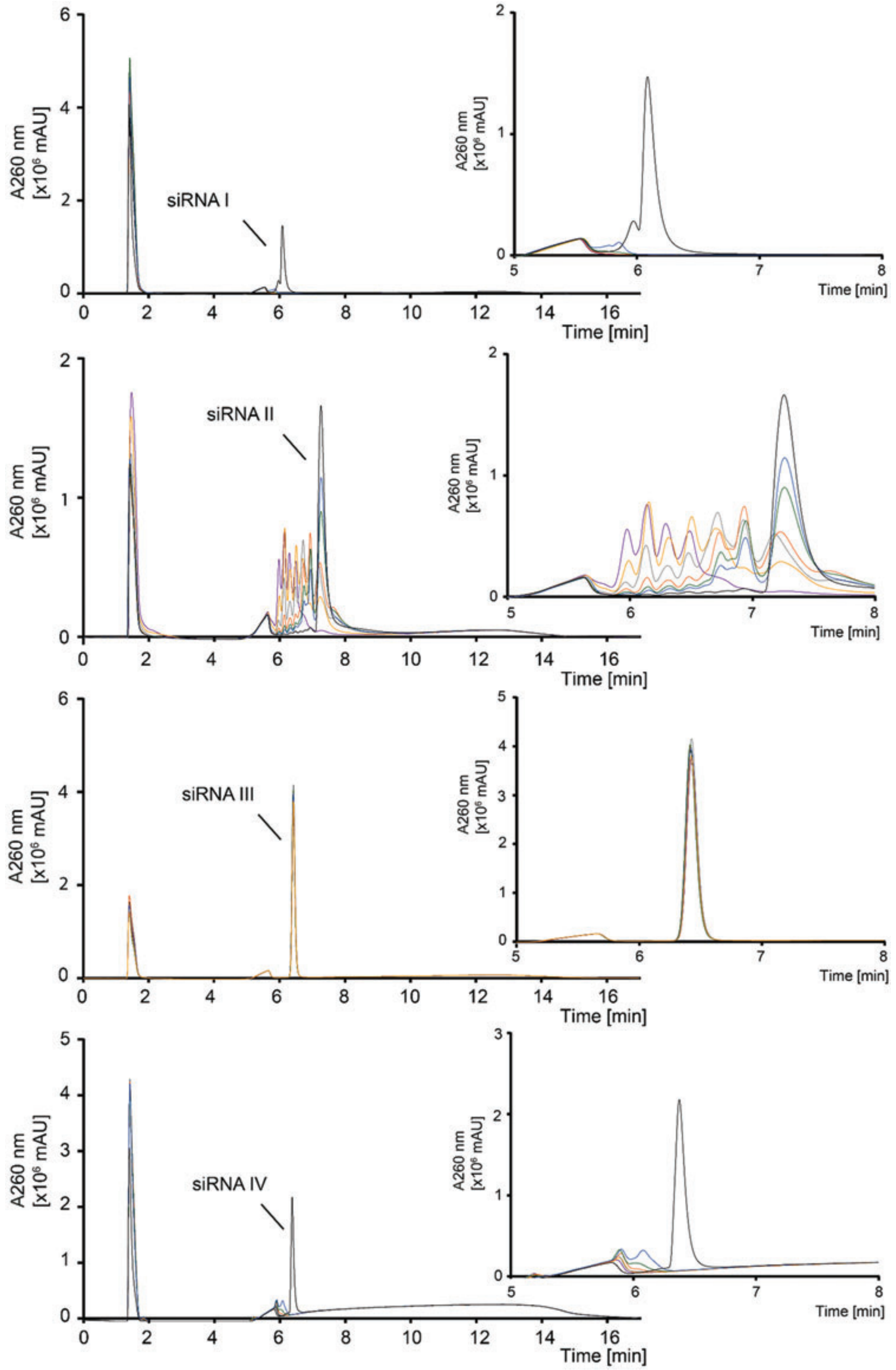

SUPPLEMENTARY FIG. S4. Representative anion-exchange HPLC chromatograms from tritosome assays. siRNAs were incubated in rat liver tritosome extracts (in 20 mM sodium-citrate buffer, pH = 5.0) for the indicated time points. Samples were analyzed on a Hitachi VWR LaChrom Elite HPLC fitted with a DNA Pac PA200 (4 × 250 mm) anion exchange column and a DNA Pac PA200 (4 × 50 mm) guard column at 30°C. UV absorption was monitored at 260 nm. The gradient was 100% A for 2 min, followed by 54% eluent B within 5 min, increase to 100% B within 2 min, hold 100% B for 1 min, switch to 100% A within 2 min, and hold 100% A for 5 min. Eluent A was a 1:1 mixture (v:v) of buffer A and ACN. Buffer A was an aqueous solution of 1 mM EDTA and 25 mM Tris HCl (pH = 8.5). Eluent B was a 1:1 mixture (v:v) of buffer B and ACN. Buffer B was an aqueous solution of 1 mM EDTA, 25 mM Tris HCl, and 1.6 M $NaClO_4$ (pH = 8.5).

(*continued*)

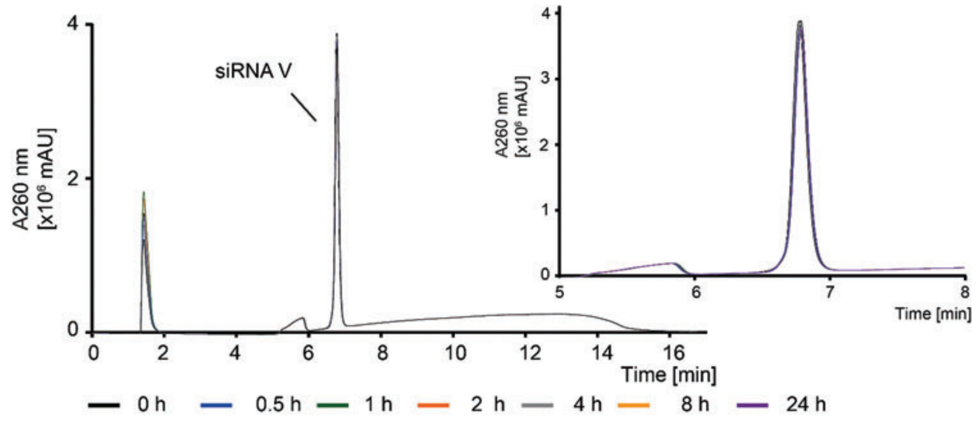

SUPPLEMENTARY FIG. S4. (*Continued*).

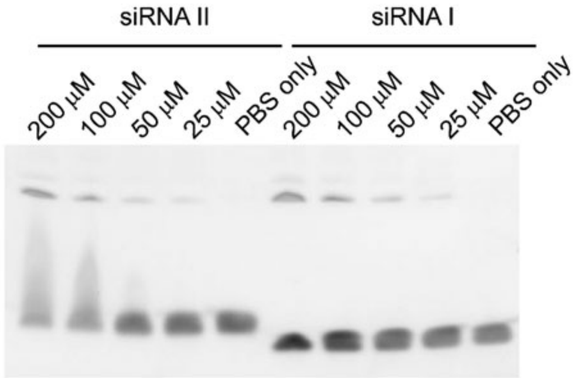

SUPPLEMENTARY FIG. S5. Albumin binding of siRNA I and II. Gel-shift assay on a 4%–20% native polyacrylamide gradient gel (60 V for 2 h) after incubation of 0.5 µM siRNAs with different concentrations of human serum albumin. Staining with SYBR Gold. Representative gel shown. $N = 3$.
