# Supplementary material for: Pharmacodynamic and Pharmacokinetic Properties of Full Phosphorothioate Small Interfering RNAs for Gene Silencing In Vivo
Source: Nucleic Acid Ther. 2021 Jun 4;31(3):237–44. doi: 10.1089/nat.2020.0852 (PMC8215415; doi:10.1089/nat.2020.0852)
Supplement: Supplemental data [file Supp_FigS6.pdf]

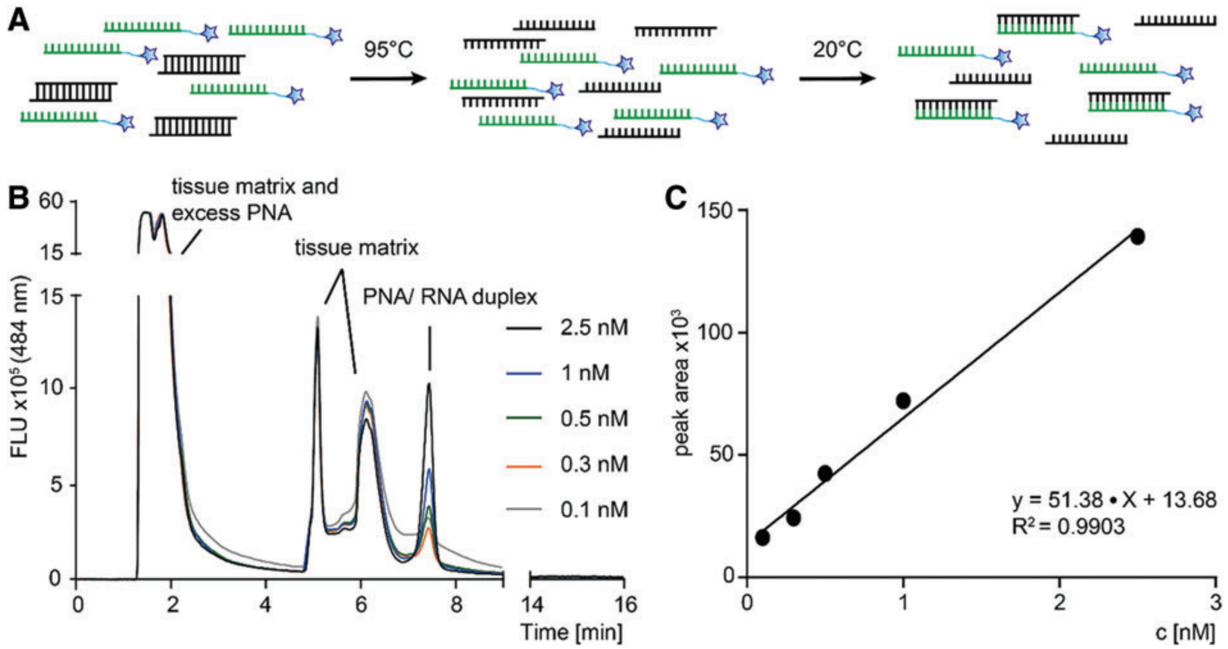

SUPPLEMENTARY FIG. S6. PNA hybridization assay for detection of siRNA II in tissue lysate. **(A)** Principle of siRNA detection with fluorescently labelled PNA probes (atto425). After heat separation of the siRNA duplex, the fluorescently labelled PNA probe anneals to the antisense strand. The RNA/PNA duplex is then analyzed by anion-exchange HPLC. **(B)** Superimposed fluorescence traces (fluorescence at 484 nm, excitation at 436 nm) of anion-exchange HPLC after spiking decreasing concentrations of siRNA II into mouse liver lysate. Analysis was performed on a Hitachi VWR LaChrom Elite HPLC fitted with a DNA Pac PA200 (4 × 250 mm) anion exchange column and a DNA Pac PA200 (4 × 50 mm) guard column at 50°C. The gradient was 100% A for 2 min, followed by 54% eluent B within 5 min, increase to 100% B within 2 min, hold 100% B for 1 min, switch to 100% A within 2 min, and hold 100% A for 5 min. Eluent A was a 1:1 mixture (v:v) of buffer A and ACN. Buffer A was an aqueous solution of 1 mM EDTA and 25 mM Tris HCl (pH = 8.5). Eluent B was a 1:1 mixture (v:v) of buffer B and ACN. Buffer B was an aqueous solution of 1 mM EDTA, 25 mM Tris HCl, and 1.6 M $NaClO_4$ (pH = 8.5). Please note that the *y*-axis scale is changed above the break. **(C)** Calibration of PNA hybridization assay in mouse liver lysate. Fluorescence peak area from **(B)** is plotted against concentration of siRNA II. PNA, peptide nucleic acid.
